# Rapid and simple detection of gero-suppressive agents

**DOI:** 10.18632/oncotarget.5455

**Published:** 2015-09-03

**Authors:** Zbigniew Darzynkiewicz

**Affiliations:** Department of Pathology, New York Medical College, Valhalla, NY, USA

**Keywords:** Gerotarget

The principal cause of organismal aging is erosion of telomeres at each cell division, which results in telomere dysfunction and leads to intrinsic (replicative) cellular senescence. Other factors, however, additionally contribute to aging [1]. Premature cellular senescence is one of them. The recent body of evidence indicates that constitutive activation of nutrient- and mitogen-signaling pathways is the primary mechanism causing cellular senescence and premature aging [2]. Activation of these pathways leads to enhanced translation, accumulation of proteins, cell growth in size and mass (hypertrophy), and cellular senescence. Particularly during the slowed down progression through the cell cycle, such as occurs during replication stress, the growth continues unabated, leading to imbalance when the ratio of protein to DNA content increases; cells grow in size and acquire the senescence (unbalanced growth) phenotype [2, 3]. The key players in this mechanism of aging are the insulin-like growth factors (IGFs) including growth hormone (GH; the “GH/IGFs axis”), AKT/PKB, and PI3K; activation along these pathways converges on mammalian target of rapamycin (mTOR) and its downstream substrate S6K that phosphorylates eIF-4B and rp-S6, the factors of initiation and continuation of translation. The evidence that inhibition of signaling along these pathways slows down the aging process and leads to increased longevity of different species organisms, including mice and rats, is undisputable [3].

Detection of anti-aging (gero-suppressive) drugs inhibiting these pathways that potentially may have application for human is of obvious importance. The quest for such modalities based on their administration to animals and an assessment of the effect on longevity, whereas the most direct, is expensive and time consuming [3]. It cannot be applied therefore for rapid screening of a variety of compounds that may have potential antiaging properties. Attempts have been made therefore to develop assays based on the *in vitro* induction of cellular senescence (gero-conversion) and its reversion to quiescence. One such tactic involves cell treatment with the drugs perturbing DNA replication to induce replication stress and assessment of the degree (“depth”) of premature cellular senescence by analysis of the classical senescence phenotype, including morphology, expression of SA-β-gal, p21^WAF1^, cyclin D1 and (00001B4)H2AX measured by imaging (laser scanning) cytometry [4-6]. The reversibility of gero-conversion by the investigated anti-aging modalities when tested in parallel with inducers of replication stress provided evidence of their potential anti-aging properties. Berberine, a natural alkaloid, that has a long history of medicinal use in both Ayurvedic and old Chinese medicine and, recently available as a dietary supplement, has been shown to have the most pronounced effect in reversing the replication stress-induced gero-conversion of A549 cells [4]. This approach, however, has not been tested as yet in terms of full reversibility of gero-conversion by assessing replicative potential of cells rescued from the replication stress by measuring their replicative capability such as clonogenicity.

In recent article Leontieva et al., [7] describe a simple, elegant, and very persuasive *in vitro* approach to detect potential anti-aging properties of ATP-competitive kinase inhibitors (Torin 1 and PP242), the agents that inhibit both mTORC1 and mTORC2. The authors examined capability of these inhibitors to suppress gero-conversion (premature senescence) induced by enforced expression of p21^WAF1^ in HT-p21 cells. The replicative potential of the cells rescued from the premature senescence by the rapamycin derivative (Deforolimus) as well as by Torin 1 and PP242 has been clearly demonstrated using a classical clonogenicity assay, whereas the irreversible cellular senescence was easily seen on parallel wells as the cytostatic effect. The ability of Torin1 and PP242 to prevent gero-conversion was also examined on WI37t fibroblasts and SkBr3 cells induced to senescence by treatment with low concentration of etoposide or doxorubicin, the agents causing replication stress. The data were supported by parallel investigation of activity β-SA-gal as well as expression of cyclin D1 and phosphorylation of rpS6 (Ser235, 236) and AKT (Ser473). The virtue of this approach, as mentioned, is its simplicity and generation of the convincing and the easy to interpret data.

A large number of supplements and other modalities advertised as having anti-aging properties are being sold through internet. Effectiveness of many of them may be questioned. Because of their sheer number, the possibility of evaluating their efficacy by testing on animals’ longevity models is limited. The rapid and relatively inexpensive *in vitro* models that can be used to evaluate competence of such products to prevent/reverse the induction of premature senescence [suppress gero-conversion] provide the alternative means to investigate validity of the advertised claims of the offered products. Since these products are of widespread use, and a subject of multibillion dollars industry, it may be advisable that independent private (e.g. Consumer Reports) or governmental (e.g. FDA) organizations would screen them using the described *in vitro* assays [4-7]. Of particular utility for such a purpose could be the simple and effective method described by Leontieva et al., [7].
